# Case of Poisoning by Nux Vomica; Recovery

**Published:** 1853-12

**Authors:** 


*Case of Poisoning by Nux Vomica; Recovery.* (Under the care of Dr. Hassall.)—Nux vomica is one of those poisonous substances for which we unfortunately possess no antidote, and whose destructive properties have, by experiments upon animals, and by accidental or wilful ingestion among human beings, been abundantly ascertained. No opportunity should, however, be lost of verifying or controlling what is known respecting the effects of nux vomica or its alkaloid strychnia, and this consideration induces us to bring the following case before our readers.

Dr. Christison states that nux vomica "is a powerful narcotic of that limited class which act almost entirely on the spinal column, producing, in poisonous doses, violent tetanic convulsions, without impairing the functions of the brain. Two drachms of the powder have proved fatal in two hours, and even *thirty grains* have been said to cause death. Those who recover from the primary effect on the nervous system may suffer from irritation in the alimentary canal, and an instance is on record of death being thus apparently produced in three days by three grains of the spirituous extract." The effects of strychnia (the proportion of the alkaloid to the nux vomica seeds being about one two-hundredth part) are stated to be as follows:—

"The slightest observable effects from small doses are twitches from the muscles of the arms and legs, occurring especially during sleep, and accompanied with restlessness, some anxiety, acceleration of the pulse, and generally slight perspiration. More rarely the bowels present increased activity, the urine is either augmented or discharged more frequently, and the venereal appetite is promoted. Larger doses cause violent startings of the muscles, or even also a tendency to locked-jaw, which are succeeded by stiffness, weariness, pain or rending in the limbs. In their highest degree these amount to violent tetanic spasm, occurring in frequent fits, with brief intervals of repose, acute sensibility, and dreadful alarm. . . . . Strychnia is one of the most subtle poisons. I have seen a wild boar killed in ten minutes with a third part of a grain of commercial strychnia injected into the cavity of the chest. I have known two-thirds of a grain cause alarming locked-jaw and general spasms in the human subject when swallowed. One grain introduced into a wound would probably prove fatal to a man; and Pelletier and Caventou have killed a dog in thirty seconds with the sixth of a grain of the pure alkaloid. . . . . *There is no antidote for it.*" Having premised thus much respecting the effects of nux vomica seeds and its alkaloid, let us describe the history and symptoms of Dr. Hassall's patient from the notes taken by Mr. Curgenven, house-surgeon to the hospital.

Abraham D——, aged twenty, a laborer of a healthy appearance, was admitted into the hospital on August 27, 1853, having three-quarters of an hour previously taken about one drachm and a half of powdered nux vomica, which he purchased for the alleged intent of poisoning rats.

When admitted he was in a profuse perspiration, the skin of the face, neck, and chest was greatly congested, the eyes suffused, the pupils slightly contracted, and the pulse hard and excited. The patient was greatly agitated, and on moving he grasped firmly the nearest object for fear of falling.

A few minutes after admission a tetanic paroxysm came on suddenly, the man was thrown into a state of opisthotonos, all his muscles becoming rigid, and respiration for the time suspended. This fit lasted about half a minute, when the muscles became relaxed, and he was again able to answer questions. Two emetics (sulphate of zinc ?) had been given him before he was brought to the hospital, but neither had acted.

On admission a sulphate of zinc emetic was administered, but without effect. The stomach-pump was then used, and mixed with what was ejected could be seen some greyish powder, but unfortunately the fluid was thrown away without any tests being used.

The patient now stated that having purchased the poison (said by the chemist to have been two drachms of powdered nux vomica) he went home, and mixed it with some water in a wine-glass, and whilst drinking it his mother knocked the glass out of his hand; he had, however, drank nearly the whole of it. Soon after the ingestion of the poison he felt a little drowsy, and the first paroxysm of tetanic spasm came on about ten minutes afterwards. He had several of these fits before he was brought to the hospital, and five after his admission into the ward. They went on decreasing in severity, and none were observed after the fifth was over.

The night following, the patient slept well, and the next day he complained of cramping pains in his limbs when he moved them; tongue rather dry; much thirst; bowels confined. He was ordered an aperient and a saline mixture.

On the 29th, the second day after admission, the pains had left him, his bowels had acted freely, the feverish symptoms had subsided, and the following day the man was discharged in very good condition.

It is a pity that the exact quantity of nux vomica powder which the patient took could not be ascertained; but it may approximatively be said that the dose was a very dangerous one, lying as it does, between the two drachms and the thirty grains mentioned by Dr. Christison. The case which we have just related presents some of the features which have been described by toxicologists—viz., tetanic spasms of a very violent nature succeeding each other very rapidly, and which disappeared completely when the poison had been washed away. Permanent locked-jaw did not, however, set in, but the cramping pains in the limbs, which came on towards the second day, and the uncertainty of gait which the patient manifested on his admission, were quite in keeping with the usual effects of the poisonous substance. The undoubted usefulness of the stomach-pump was well-shown in this case, and the circumstance affords an additional proof that this valuable instrument is one of the best contrivances for thoroughly emptying and washing out the cavity of the stomach.—*London Lancet.*

